# Antagonists of the Mu-Opioid Receptor in the Cancer Patient: Fact or Fiction?

**DOI:** 10.1007/s11912-022-01295-z

**Published:** 2022-06-01

**Authors:** Amparo Belltall, Guido Mazzinari, Oscar Diaz-Cambronero, Pilar Eroles, María Pilar Argente Navarro

**Affiliations:** 1 Research Group in Perioperative Medicine, Hospital Universitario y Politécnico la Fe, Valencia, Spain; 2 Department of Anaesthesiology, Avenida de Fernando Abril Martorell, 106, 46026 Valencia, Spain; 3 Euro-Periscope, Onco-Anaesthesiology Research Group (RG) of European Society of Anaesthesiology and Intensive Care, Valencia, Spain; 4 INCLIVA Biomedical Research Institute, Valencia, Spain Avenida de Menéndez y Pelayo, 4, 46010 Valencia, Spain; 5 University of Valencia INCLIVA-Hospital Clínico de Valencia-CIBERONC, Valencia, Spain; 6 Department of Medical Oncology, Avenida de Menéndez y Pelayo, 4, 46010 Valencia, Spain

**Keywords:** PAMORAs, Peripheral antagonists of mu-opioid receptor, Methylnaltrexone, Naltrexone, Low-dose naltrexone, Cancer, Tumor progression, Cancer growth, Opioid growth factor (OGF), Opioid growth factor receptor (OGFr), Mu-opioid receptor (MOR)

## Abstract

**Purpose of Review:**

Antagonists of mu-opioid receptor role in cancer progression remains to be elucidated. The objective of this review was to summarize the available evidence on antagonists of mu-opioid receptor effect on tumor progression and prognosis in different types of cancers and an evaluation of the available findings on their mechanism of action.

**Recent Findings:**

We have found studies related to methylnaltrexone (MNTX) and naltrexone (NTX) usage in cancer outcomes-related setting. We found consistent preclinical evidence of a potential action of MNTX and NTX on cancer growth and spread mediated mainly by effect on the opioid growth factor receptor (OGFr) axis, which results in depressed cell replication. However, clinical results are scarce and limited to poor-quality evidence.

**Summary:**

Further high-quality studies are warranted to study antagonists of mu-opioid receptor role as a therapeutic option in different types of cancer, especially in patients where the classical treatment causes unacceptable side effects.

## Introduction

Opioids are cornerstone in the management of perioperative pain, but their use is also linked to potential worse oncological outcomes [1]. Mu-opioid receptor (MOR) is overexpressed in many cancer types versus normal tissue[1–10] and its activation appears to facilitate VEGF induced angiogenesis [11•, 12], to increase vascular permeability and to blunt immune response [13, 14]. However, the role of opioids and MOR in tumor progression is still debated [15] due to the flawed design of numerous preclinical studies [13]. It is believed that antagonists of mu-opioid receptor may play a role in cancer progression since MOR activation is linked to tumor progression and MOR antagonism has been postulated as a potential target strategy for cancer treatment [11•, 16–18].

There are two types of mu-opioid receptor antagonists, with a central effect, such as naltrexone (NTX) and only peripherally acting mu-opioid receptors antagonists (PAMORAs). The PAMORAs are specifically designed to avoid blood–brain barrier penetration and counteract the mu-opioid-related side effects outside the central nervous system (CNS)[19] had been studied in postoperative nausea and vomiting (PONV) and postoperative ileus (POI) [20, 21, 22•, 23, 24]. Their current main therapeutic indication is opioid-induced constipation (OIC) related to MOR activation in the gastrointestinal tract mainly in patients receiving chronic opioid therapy, considered safe, effective, and well-tolerated agents [25–27, 28•].

Nonetheless, mu-opioid receptor antagonists can influence cancer progression through alternative pathways as the interaction with opioid growth factor-opioid growth factor receptor (OGF-OGFr). OGF, a chemically termed [Met5]-enkephalin, is an endogenous pentapeptide with potential antineoplastic and antiangiogenic activities that binds to and activates the OGFr, present on some tumor cells and vascular cells, thereby inhibiting tumor cell proliferation and angiogenesis [29, 30•, 31]. The immune system can also be regulated by controlling the expressions of endocrine system signaling molecules. The binding of OGF to kappa and delta opioid receptors (KOR and DOR) on immune cells, rather than MOR, influenced immune regulation [30•].

Therefore, the objective of this review is to summarize the available evidence on antagonists of mu-opioid receptor effect on tumor progression and prognosis in different types of cancers and an evaluation of the available findings on their mechanism of action.

## Methods

A literature search was conducted using PubMed with a January 2000 to December 2021 timeframe. We restricted the search to English language. Several MeSH-based searches were carried out in Pubmed using the keywords “Peripherally acting μ-opioid receptor antagonists” OR PAMORA OR Naltrexone OR Methylnaltrexone OR Naloxegol OR Alvimopan OR Naldemedine OR Nalmefene for cancer. We included original studies where MOR antagonists were used at any dosage, focusing on cancer of any type, reporting on tumor size, growth, clinical progression, or oncologic-related outcomes in perioperative medicine as defined by a recent consensus paper [32]. Preclinical and clinical studies with prospective or retrospective observational data collection or randomized clinical trials were included. Studies in which MOR antagonists were used concurrently with chemotherapy or other drugs were not excluded, but details of each concurrent therapy were noted. Editorials, reviews, and abstracts were excluded. Two authors (AB and GM) independently carried out the selection process, and disagreements were resolved by a third author (ODC). A total of 7115 articles were obtained. We screened all titles and selected a subset of articles for full abstract review. After the abstract review, we selected 110 articles for full-text review and screened the bibliography for additional interesting articles. Finally, we included in the review 23 articles that were related to cancer. We report preclinical investigations in Table 1 and clinical investigations in Table 2. Due to the paucity of data we retrieved, we were only able to carry out a narrative review.

**Table 1:** Preclinical studies assessing the effect on PAMORAs on cancer-related outcomes

Title:	Participants:	Drugs, dosages and treatment period:	Available outcome:
The novel role of the mu-opioid receptor in lung cancer progression: a laboratory investigation [18].	Human NSCLC cell lines H522, H1703, H1993, SW1573, H1437, H358, control BEAS-2B, and mouse LLC cells were obtained from ATCC (Walkersville, MD) C57BL/6 and MOR knockout mice. All mice were 8- to 12-week-old females obtained from Jackson Laboratories (Bar Harbor, ME)	MNTX 0, 10, or 100 nM or MOR shRNA. Morphine and DAMGO	MOR agonists (morphine and DAMGO) increased LLC cell growth in vitro. MNTX or silencing MOR expression inhibited LLC invasion and anchor-independent growth. Injection of MOR-silenced LLC leads to a reduction in mouse lung metastasis. MOR knockout mice do not develop significant tumors when injected with LLC compared to wild-type controls. Continuous infusion of MNTX attenuates primary LLC tumor growth and reduces lung metastasis
Nalmefene attenuates malignant potential in colorectal cancer cell via inhibition of opioid receptor [33].	Murine CT26 colon cancer cells	Nalmefene was administered to cells in a concentration gradient (N1: 0.625 μg/l, N2: 0.25 μg/l, N3: 1 μg/l, and N4: 10 μg/l) for 10 h	Nalmefene inhibited CT26 cell viability and migration in a concentration-dependent manner, also inhibited glycolysis of CT26 cells. The antitumor effect can be achieved through opioid receptor inhibition and downregulation of calmodulin expression and CaMK II phosphorylation, thus inhibiting the AKT-GSK-3β pathway and CT26 cell glycolysis
Low doses of methylnaltrexone inhibits head and neck squamous cell carcinoma growth in vitro and in vivo by acting on the mu-opioid receptor [34].	Human HNSCC cell lines: FaDu (from the American Type Culture Collection), MDA686Tu and UMSCC47 (from the laboratory of Dr. Jeffrey N. Myers, The University of Texas M.D. Anderson Cancer Centre) Six- to seven-week-old male athymic nu/nu mice (C57BL/6) were purchased from the Envigo Harlan Laboratories	DAMGO: 10 nM, 100 nM and 1 μM MNTX: 1 nM, 10 nM, 100 nM and 1 μM for 24,48 and 72 h MNTX 1,10 and 100 μg/kg/day or placebo (0.9% saline solution) were randomly assigned to each mouse	FaDu and MDA686Tu cell lines express MOR and UMSCC47 not express the receptor. Activation of the receptor with DAMGO caused a significant reduction in cAMP levels in FaDu cells. Knockdown of MOR inhibited in vitro aggressive cell behaviors on FaDu and MDA686Tu cells. MNTX strongly inhibited the proliferation, clonogenic activity, invasion and migration of FaDu and MDA686Tu cells but has no effect on UMSCC47 cells. In vivo MNTX suppresses tumor growth in HNSCC cell tumor-bearing mice
Low-dose naltrexone suppresses ovarian cancer and exhibits enhanced inhibition in combination with cisplatin [38].	The human ovarian cancer cell line SKOV-334 was obtained from The American Type Culture Collection (Manassas,VA, USA) Four-week-old athymic nu/nu female mice, purchased from the Charles River Laboratory (Wilmington, MA, USA),	NTX (1025 mol/L), taxol (1029 or 10,210 mol/L), cisplatin (0.01 or 0.001 mg/mL), NTX (1025 mol/L) and taxol (1029 or 10,210 mol/L), NTX (1025 mol/L) and cisplatin (0.01 or 0.001 mg/mL), or an equivalent volume of sterile water	Reduced DNA synthesis and cell proliferation. Enhanced anticancer action. Tumor progression in a non-toxic fashion by reducing DNA synthesis and angiogenesis. Upregulated expression of the OGF and OGFr
The opioid growth factor (OGF) and low-dose naltrexone (LDN) suppress human ovarian cancer progression in mice [39].	Female nude mice were transplanted intraperitoneally with SKOV-3 human ovarian cancer cells	OGF (10 mg/kg), LDN (0.1 mg/kg), or an equivalent volume of vehicle (saline). 40 days of treatment	OGF and LDN markedly reduced ovarian tumor burden (tumor nodule number and weight). The mechanism of action was targeted to an inhibition of tumor cell proliferation and angiogenesis; no changes in cell survival were noted
Beta 2 Adrenergic Receptor Antagonist Propranolol and Opioidergic Receptor Antagonist Naltrexone Produce Synergistic Effects on Breast Cancer Growth Prevention by Acting on Cancer Cells and Immune Environment in a Preclinical Model of Breast Cancer [40].	Human breast cancer cells MDA-MB-231 (RRID:CVCL_0062), MDA-MB-468 (RRID: CVCL_0419), and T47D (RRID:CVCL_0553) were obtained from American Type Culture Collection (ATCC; Rockville, MD, USA), and MDA-MB-231/Luc-GFP cells were purchased from GenTarget Inc T-cell-deficient, athymic nude (Crl:NIH-Foxn1rnu) female rats aged 21–28 days old were purchased from Charles River (Portage, MI, USA)	PRO and NTX alone or in combination with increasing concentrations (0.001 μM to 200 μM) for 24, 48, and 72 h	Inhibited the cell growth and progression of breast cancer cells in vitro in cultures, and in vivo in rat xenografts
Synergistic effects of methylnaltrexone with 5-fluorouracil and bevacizumab on inhibition of vascular endothelial growth factor-induced angiogenesis [41].	HPMVEC	MNTX, 5-FU, 10 nM MNTX + 5-FU, 100 nM MNTX + 5-FU, bevacizumab, 10 ng/mL MNTX + Bevacizumab, 50 ng/mL MNTX + Bevacizumab, Naltrexone, 10 nM Naltrexone + 5-FU, 50 nM Naltrexone + 5-FU, 10 ng/ml Naltrexone + Bevacizumab, 50 ng/ml Naltrexone + Bevacizumab	MNTX exerts a synergistic effect with 5-FU and bevacizumab on inhibition of VEGF-induced human pulmonary microvascular EC proliferation and migration. These synergistic effects were not observed with naltrexone
Methylnaltrexone, a Peripherally Acting Opioid Receptor Antagonist, Enhances Tumoricidal Effects of 5-FU on Human Carcinoma Cells [44].	The SW-480 colorectal cancer cells (Leibovitz’s L-15), MCF-7 breast cancer cells (RPMI-1640) and non-small cell lung cancer cells (DMEM) were obtained from the American Type Culture Collection (ATCC, Manassas, VA, USA)	5-FU 10 μM alone, 5-FU 10 μM plus MNTX 0.01 or 0.1 or 1.0 μM. MNTX alone (0.01, 0.1 and 1.0 μM)	MNTX enhanced the actions of 5-FU. MNTX alone also showed antiproliferative activity in all three cell lines. MNTX increased cell number in the G1 phase and decreased cyclin A expression
Low-dose naltrexone inhibits colorectal cancer progression and promotes T apoptosis by increasing M1-type macrophages and activating the Bax/Bcl-2/caspase-3/PARP pathway [45].	The human colon cancer cell lines SW480 and HCT116 were obtained from the Cell Bank of the Chinese Academy of Sciences. Normal human colon cells (NCM460 cells, Cell Bank, Chinese Academy of Sciences) were used as controls Female BALB/c nude mice (4–6 weeks old) were purchased from Beijing Vital River Laboratory Animal Technology Co., Ltd. (Beijing, China)	Cells were incubated with 0.25, 0.5, 1, 1.5, and 2 mg/mL of LDN (dissolved in RPMI 1640 prior to addition) for 24, 48, and 72 h 2 groups (5 mice in each group), including the LDN treatment group (intraperitoneal (i.p.) 5 mg/kg/2 days LDN) and normal saline (NS) group	Tumor size was measured using a caliper. Tumor volume was calculated according to the following formula: volume (mm3) = (length × width2)/2
Low-dose naltrexone inhibits the epithelial-mesenchymal transition of cervical cancer cells in vitro and effects indirectly on tumor-associated macrophages in vivo [46].	Hela, Siha, C33A and Caski human cervical cancer cell lines were purchased from the Cell Bank of the Chinese Academy of Sciences ALB/C nude mice (4–6 weeks old) were purchased from Charles River (Beijing, China)	LDN was dissolved in DMEM to 0.5, 1.5, 2, 3, and 5 mg/mL, and the culture was continued for 24, 48, and 72 h The mice were randomly divided into four groups with five animals in each group, including the control group, the 0.5 mg/kg group, the 5 mg/kg group, and the 10 mg/kg group based on the concentrations of LDN administered to the mice every day	Suppressed the proliferation, migration and invasion abilities and promote apoptosis in Hela cells Inhibit cervical cancer progression in nude mice. Reduced the number of TAMs, mainly M2 macrophages, and decreased expression of anti-inflammatory factor IL-10 in the serum of nude mice
Low-dose naltrexone plays antineoplastic role in cervical cancer progression through suppressing PI3K/AKT/mTOR pathway [47].	Human cervical cancer cell lines Hela and Siha (TCHu187 and TCHu113) were purchased from the Cell Bank of the Chinese Academy of Sciences 4 × 106 Hela cells (in 0.1 ml solution) were injected into BABL/c nude mice. The nude mice experiments were performed in four independent groups	For treatment with LDN, medium containing 1.24 (Hela) or 1.83 (Siha) mg/mL LDN (Amquar Bio Co., Ltd, USA) was added Different dose of LDN were intraperitoneally injected as 0.5 mg/kg, 5 mg/kg, 10 mg/kg	Upregulate the expression of OGFr. Suppress the abilities of colony formation, migration and invasion in cervical cancer cells. Inhibit cervical cancer progression in mice model. Indirectly reduced the expressions of PI3K, pAKT and mTOR in vitro and in vivo
Peripheral Opioid Antagonist Enhances the Effect of Anti-Tumor Drug by Blocking a Cell Growth-Suppressive Pathway In Vivo [48].	Gastric cancer tissues and patient’s ascites was provided by the National Cancer Center Hospital. Different human gastric cancer cell lines (HSC-60, 60As6, HSC-39, HSC-42, HSC-43, HSC-44, 44As3, HSC-58, 58As1, 58As9, HSC-59, SNU-16, KATOIII, MKN45, TMK-1, OKAJIMA, PANC-1) and mouse fibroblast cell line (NIH3T3) were used Six-week-old female C.B17/Icr-scid mice were purchased from CLEA Japan (Tokyo, Japan)	[Met5]-enkephalin (OGF) was purchased from Wako (Tokyo, Japan) (10^−4^ M). Methylnaltrexone (MNTX) was provided from Drs. H. Nagase & T. Suzuki, (10^–6^ and 10^−5^ M). OGF and MNTX were dissolved in sterile water or saline (10^−6^ M). Docetaxel (Doc) was purchased from Aventis Pharma Co., Ltd. (Tokyo, Japan) (10^−9^ M). Combination of Doc + MNTX (10^−6^ M)	Doc + MNTX significantly prolongs survival, alleviates abdominal pain, and diminishes Doc-resistant spheroids on the peritoneal membrane in model mice
Modulation of the opioid growth factor ([Met(5)]-enkephalin)-opioid growth factor receptor axis: novel therapies for squamous cell carcinoma of the head and neck [49].	Nude mice with visible human SCCHN SCC-1 tumors	Exogenous OGF 10 mg/kg, OGF either once a week (1x/week OGF), 3 times weekly (Monday, Wednesday, Friday) (3x/week OGF), or daily (daily OGF) Imiquimod (5%, Aldara Cream, 3 M, St. Paul, MN) once weekly (1x/week) or 3 times weekly (Monday, Wednesday, Friday; 3x/week) LDN 0.1 mg/kg naltrexone once a week (1x/week LDN), 3 times weekly (Monday, Wednesday, Friday) (3x/week LDN), or daily (daily LDN)	OGF and LDN increased the latency from visible to measurable tumors up to 1.6-fold. OGF, LDN, and imiquimod treatment markedly reduced tumor volume and weight, and decreased DNA synthesis in tumors
Opioid antagonists inhibit the growth of metastatic murine neuroblastoma [50].	S2OY neuroblastoma (NB) in A/Jax mice	Daily injections of 0.1 mg/kg NTX	69% tumor take, 70% delay in time prior to tumor appearance, and a 60% increase in median survival time. The pattern and incidence of metastases of NTX and control mice were similar
Successive treatment with naltrexone induces epithelial–mesenchymal transition and facilitates the malignant biological behaviors of bladder cancer cells [51].	The T24 human bladder cancer cell line and MB49 mouse bladder cancer cell line were obtained from the FuHeng Cell Center (Shanghai, China)	Different concentrations (1, 10, and 100 μM) of naltrexone (Aladdin, Shanghai, China) were added to the medium for incubation for 24 h	Successive treatment with naltrexone may be favorable for the progression of bladder tumors by activating the PI3K/AKT signaling pathway and inducing EMT

PAMORAs: Peripheral antagonist of mu-opioid receptor; NSCLC: non-small cell lung cancer; DAMGO: D-Ala(2), N-Me-Phe(4), Gly(5)-ol-enkephalin; cAMP: cyclic adenosine monophosphate; HNSCC: head and neck squamous cell carcinoma; VEGF: vascular endothelial growth factor; HPMVEC: Human pulmonary microvascular endothelial cell; EC: endothelial cell; mTOR: mammalian target of rapamycin; LPS: Lipopolysaccharide; SCCHN: squamous cell carcinoma of the head and neck; MNTX: Methylnaltrexone; 5-FU: 5-fluorouracil; mTOR: mammalian target of rapamycin; CaMK II: calcium/calmodulin dependent protein kinases II; AKT-GSK-3β: serine/threonine kinase (AKT)-glycogen synthase kinase-3β (GSK-3β); PRO: propranolol; NTX: naltrexone; LDN: low-dose naltrexone; DMEM: Dulbecco’s modified Eagle’s medium; TAMs: tumor-associated macrophages; OGF: opioid growth factor; OGFr: opioid growth factor receptor; LLC: Lewis lung carcinoma; Doc: docetaxel; EMT: epithelial-mesenchymal transition

**Table 2:** Clinical studies assessing the effect on PAMORAs on cancer-related outcomes

Title:	Participants:	Drugs, dosages and treatment period:	Available outcome:
Treatment with methylnaltrexone is associated with increased survival in patients with advanced cancer [52•].	229 patients with advanced end-stage cancer and 134 patients with other advanced illnesses, who were treated with regular opioids and experienced OIC despite laxatives, enrolled in two placebo-controlled, double blinded multicenter randomized clinical trials (NCT00401362 and NCT00672477)	MNTX or placebo was given subcutaneously during the double-blinded phase, which was followed by the open label phase, allowing MNTX treatment irrespective of initial randomization	Treatment with MNTX and even more so response to MNTX are associated with increased OS in patients with advanced end-stage cancer. There was no difference in OS between MNTX and placebo in the 134 patients with advanced illness other than cancer
A new neuroimmunotherapeutic strategy of subcutaneous low-dose interleukin-2 plus the long-acting opioid antagonist naltrexone in metastatic cancer patients progressing on interleukin-2 alone [53].	Ten patients with metastatic renal cancer, who had progressed on a previous immunotherapeutic cycle with IL-2 alone	Patients were treated with the same doses of IL-2 (6 million IU/day subcutaneously for 6 days/week for 4 weeks) plus an oral administration of NTX at a dose of 100 mg every 2 days	The clinical response consisted of a partial response in 1 and a stable disease in 5 patients, whereas the other 4 patients progressed. Therefore, the percent of non-progressive disease was 6/10 (60%). Mean lymphocyte increase achieved during IL-2 plus NTX was significantly higher than that obtained during the previous treatment with IL-2 alone
Low-Dose Naltrexone and Lung Cancer: A Case Report and Discussion [54].	A 50-year-old male with T3, N1, Mx lung cancer and a history of prostate cancer. The patient underwent surgery, performing an upper right lobectomy + chemotherapy, which was later rejected due to intolerable side effects. However, he did complete the radiation regimen	LDN 4.5 mg HS	Prolonged survival
Long-term remission of adenoid cystic tongue carcinoma with low-dose naltrexone and vitamin D3-a case report [55].	A 58-year-old male with adenoid cystic tongue carcinoma	LDN was started at a dose of 3 mg orally once daily at bedtime, subsequently increased to 4 mg + oral vitamin C + high-dose vitamin D	Long-term remission with absence of active disease in MRI
The long-term survival of a patient with pancreatic cancer with metastases to the liver after treatment with the intravenous alpha-lipoic acid/low-dose naltrexone protocol [56].	A 46-year-old man diagnosed with poorly differentiated adenocarcinoma of the pancreas with metastases to the liver	Intravenous alpha-lipoic acid 300 to 600 mg 2 days per week and low LDN oral 4.5 mg HS (ALA/N). Triple antioxidant regimen consisting of oral ALA (600 mg/d), selenium (200 mg 2 times per day), and silymarin (300 mg 4 times a day) was added	Long-term survival, free from symptoms, and without appreciable progression of his malignancy
Revisiting the ALA/N (alpha-lipoic acid/low-dose naltrexone) protocol for people with metastatic and nonmetastatic pancreatic cancer: a report of 3 new cases [57].	Three pancreatic cancer case: - A 74-year-old woman who was diagnosed with mucinous adenocarcinoma of the pancreas with metastases to the liver - A 80-year-old woman with adenocarcinoma of the pancreas with possible metastatic disease of the liver - A 67-year-old male, in addition to his pancreatic cancer with liver and retroperitoneal metastases, had a history of B-cell lymphoma and prostate adenocarcinoma	Intravenous alpha-lipoic acid 300 to 600 mg 2 days per week and LDN oral 4.5 mg HS (ALA/N). Triple antioxidant regimen consisting of oral ALA (600 mg/d), selenium (200 mg 2 times per day), and silymarin (300 mg 4 times a day) was added	Long-term survival and no signs of pancreatic disease
Reversal of signs and symptoms of a B-cell lymphoma in a patient using only low-dose naltrexone [58].	A 61-year-old man with B-Cell lymphoma	LDN (3 mg every night at bedtime), previously he completed 9 treatments with intravenous α-lipoic acid (ALA) in the first week	The patient reported being asymptomatic and improvement was evidenced on examination and on complementary imaging tests of his lymphadenopathy
The Long-Term Survival of a Patient With Stage IV Renal Cell Carcinoma Following an Integrative Treatment Approach Including the Intravenous α-Lipoic Acid/Low-Dose Naltrexone Protocol [59].	A 64-year-old male patient diagnosed with metastatic renal cell carcinoma. In spite of a left nephrectomy and the standard oncological protocols, the patient developed a solitary left lung metastasis that continued to grow	Initially IV vitamin C 25 to 50 g every morning and IV racemic ALA 300 to 600 mg every afternoon The oral protocol included LDN 4.5 mg at bedtime, the oral Triple Antioxidant Therapy protocol with racemic ALA 300 mg twice daily, selenomethionine 200 μg twice daily, and silymarin 900 mg twice a day along with 3 professional-strength B-50 complex capsules a day. Oral hydroxycitrate (HCA) 500 mg times daily was added to the protocol	The patient had stable disease with disappearance of the signs and symptoms of stage IV RCC, a full 9 years following diagnosis, with a gentle integrative program, which is essentially free of side effects
Metabolic treatment of cancer: intermediate results of a prospective case series [60].	Ten consecutive patients with chemoresistant advanced metastatic cancer were offered compassionate metabolic treatment. Primary sites were lung carcinoma (n = 2), colonic carcinoma (n = 2), ovarian carcinoma (n = 1), esophageal carcinoma (n = 1), uterine sarcoma (n = 1), cholangiocarcinoma (n = 1), parotid carcinoma (n = 1) and unknown primary (n = 1). The patients had been heavily pretreated. An eleventh patient with advanced prostate cancer resistant to hormonotherapy is also reported	They were treated with a combination of lipoic acid at 600 mg iv, hydroxycitrate 500 mg t.i.d. and LDN 5 mg at bedtime The patient with advanced prostate cancer resistant to hormonotherapy received treatment with hydroxycitrate, lipoic acid and antiandrogen	Two patients died of progressive disease within two months. Two other patients had to be switched to conventional chemotherapy combined with metabolic treatment, one of whom had a subsequent dramatic tumor response. The disease in the other patients was stable or very slowly progressive. The hormone-resistant prostate cancer patient had a dramatic drop in PSA (90%)

MRI: Magnetic Resonance Imaging; IV: intravenous; LDN: Low-dose naltrexone; MTNX: methylnaltrexone; NTX: Naltrexone; PSA: prostate specific antigen; OS: overall survival; HS: hour of sleep; ALA: alpha-lipoic acid

## Antineoplastic Mechanism of Action

**Fig. 1:**
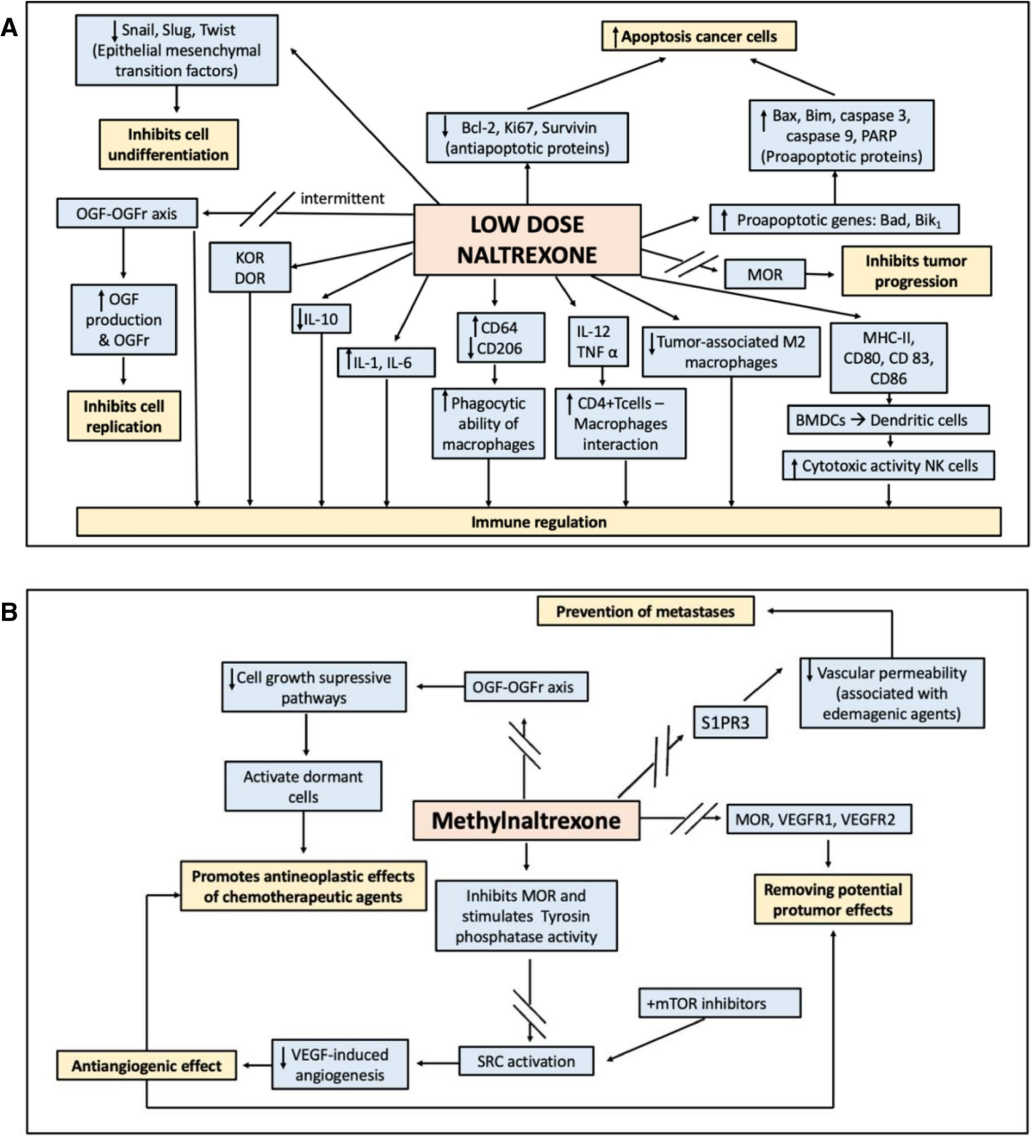
Antagonists of mu-opioid receptor biochemical mechanisms of antineoplastic action. **A**: Low-dose naltrexone biochemical mechanisms of antineoplastic action. **B**: Methylnaltrexone biochemical mechanisms of antineoplastic action. OGF: opioid growth factor; OGFr: opioid growth factor receptor; KOR: κ opioid receptor; DOR: δ opioid receptor; MOR: µ opioid receptor; BMDCs: bone marrow-derived dendritic cells; S1PR3: sphingosine-1-phosphate receptor 3; mTOR: mammalian target of rapamycin;  : inhibitory effect

The mechanism of the potential antitumor effect of MOR antagonists has been investigated primarily using NTX and methylnaltrexone (MNTX), although we found a single article discussing the effect of nalmefene on cellular glycolysis [33]. There is a dual ambivalent effect of MOR antagonists, mainly studied with NTX on cancer cells and immunity depending on dosage [34]. The effects of low-dose NTX and MNTX are summarized in Fig. 1.

NTX exhibits a dose-dependent dual immunoregulator effect on cell proliferation in vivo and in vitro[35, 36•]. It has been reported that intermittent blockade by low-dose NTX can result in a feedback production of more opioid peptides and receptors, and thus inhibiting cell proliferation via compensatory up-regulation of OGF and OGFr. However, continued blockade can suppress the activity of OGFr.^15^ NTX dosages are different depending on indications. While NTX is used for drug withdrawal and prevention of relapse at the dosage of 50 mg/day, it can be used to regulate chronic pain and treat immune diseases at the dosage of 5 mg/day, which is defined as low-dose NTX [30•]. Low-dose NTX carries out an intermittent OGFr blockade and OGF-OGFr axis upregulation activation which inhibits cell replication and has been reported to play a role in reducing tumor progression, [36•] whereas higher NTX doses cause continuous OGFr blockade, which results in enhanced cell growth [37]. The OGF-OGFr axis may be targeted for cancer treatment by (I) administration of exogenous OGF, (II) genetic manipulation to overexpress OGFr, and (III) use of low-dose NTX to stimulate OGF-OGFr axis after intermittent receptor blockade. The OGF-OGFr axis has been proposed as a therapeutic target (I) prophylactically, (II) after surgical debulking, or (III) in conjunction with standard chemotherapy for additional efficacy [38, 39].

Furthermore, tumor growth can be reduced by regulating the function of the immune system by low-dose NTX through several mechanisms. First, low-dose NTX can increase the phagocytic ability of macrophages. In addition, low-dose NTX can increase the secretion of various cytokines such as IL-1 and IL-6.^20^ Low-dose NTX can also increase the interactions between CD4 + T cells and macrophages. Moreover, it promotes the maturation of bone marrow-derived dendritic cells (BMDCs) to dendritic cells and can stimulate the cytotoxic activity of NK cells [30•]. Besides, low-dose NTX administration increases the proapoptotic expression of the genes Bad and Bik_1_ and enhances cells sensitivity to the cytotoxic effects of various standard chemotherapy agents [35]. Other proapoptotic effects include Bax and p-Bax, p-Bim, caspase 3 and cleaved caspase 3 levels increase, and Bcl-2 downregulation. In addition, a NTX-related reduction in the production of Snail, Slug and Twist epithelial-mesenchymal transition factors by tumor cells was also demonstrated [40].

On the other hand evidence from in vitro studies shows that NTX blocks MOR, VEGFR1 and VEGFR2 activation in a concentration-dependent manner, thus removing their potential protumor effect.^12^ Also, MNTX enhances the angiogenesis inhibition properties of various chemotherapy agents such as 5-fluorouracil (5-FU), bevacizumab, docetaxel, temsirolimus, or rapamycin in human pulmonary microvascular endothelial cells (ECs). This synergistic effect was not observed with NTX [41, 42].

Other in vitro and in vivo data have suggested that pretreating with MNTX can blunt the increased vascular permeability associated with the administration of edemagenic agents like lipopolysaccharide (LPS), thrombin, and MOR agonists such as morphine or D-Ala(2), N-Me-Phe(4), Gly(5)-ol-enkephalin (DAMGO) in pulmonary ECs and murine lungs. MNTX provides barrier protection against edemagenic agents by inhibiting sphingosine-1-phosphate receptor 3 (S1PR3) activation. Maintenance of this barrier could play a role in the prevention of metastases [43].

## Effect on Oncologic Outcomes

We have not found studies related to alvimopan, naloxegol, or naldemedine usage in cancer outcomes-related settings. Most of the information found refers to use of MNTX and NTX, although an article refers to nalmefene [33].

### Preclinical Evidence

Numerous articles assessed MOR antagonists role in tumor growth and spread in different tumor cell lines and animal models both as a standalone treatment or in combination with some chemotherapy agent, such as 5-FU [41, 44].

A study carried out in a human breast cancer cellular model and in a murine xenograft showed how the administration of β2 adrenergic blocker propranolol and NTX, inhibit the cell growth, colony formation, migration, invasion, and cell cycle progression of MDA-MB-231, MDA-MB-468, and T47D cells lines in vitro. The antitumor effect was enhanced by propranolol and NTX combined treatment. In addition, in vivo tumor growth was reduced and the survival time of the animal increased [40].

Another study explored the mechanisms underlying low-dose NTX inhibitory effect on the progression of colorectal cancer (CRC) in vivo and in vitro*,* suggesting that it can reduce tumor size. The authors found that low-dose NTX reduces CRC tumor size by increasing M1 macrophages and tumor necrosis factor-α (TNF-α). Also, low-dose NTX was able to upregulate OGFr expression and the apoptosis-related factors Bax, caspase-9, caspase-3 and PARP and down-regulate the expression of Bcl-2 and Ki67 to promote tumor cell apoptosis [45].

The effects of low-dose NTX have also been investigated on the epithelial-mesenchymal transition of cervical cancer cells in vitro and its influence on macrophage polarization and associated cytokines in vivo. The published results suggested that low-dose NTX suppressed proliferation, migration, and invasion capabilities and promoted apoptosis in Hela cells, a human cervical cell line. When OGFr was knocked out, the effect of low-dose NTX on the inhibition of the epithelial-mesenchymal transition of cervical cancer cells was weakened. Low-dose NTX inhibits cervical cancer progression in nude mice. Furthermore, low-dose NTX indirectly reduced the number of tumor-associated M2 macrophages and decreased the expression of anti-inflammatory factor IL-10 in the serum of nude mice [46]. The same authors postulate that low-dose NTX could upregulate the expression of OGFr. Furthermore, low-dose NTX indirectly reduced the expressions of phosphatidylinositol-3-kinase (PI3K/AKT), pAKT and mTOR in vitro and in vivo [47].

Concerning head and neck squamous cell carcinoma (HNSCC), in vitro studies showed that MNTX strongly inhibited the proliferation, clonogenic activity, invasion and migration of two HNSCC cell lines (FaDu and MDA686Tu), but has no effect on UMSCC47 cells. In vivo experiments demonstrated that MNTX suppresses tumor growth in HNSCC cell tumor-bearing mice [34].

A recent flow cytometry study tested the apoptotic effect of MNTX in combination with 5-FU on human SW-480 CRC cells, MCF-7 breast cancer cells, and non-small cell lung cancer (NSCLC) cells [44]. 5-FU significantly decreased cancer cell growth in all three cell lines in a concentration-dependent manner, and MNTX enhanced the 5-FU effect. MNTX alone also showed antiproliferative activity although it did not induce apoptosis in any of the three cell lines. Therefore, MNTX at therapeutic concentrations for OIC does not attenuate and can improve 5-FU tumoricidal activity. The enhanced activity of 5-FU can be attributed to the different pathways of 5-FU and MNTX exerting a synergistic effect. This effect could give MNTX a complementary role in treating cancer with chemotherapeutic agents [44].

In a different study, Lewis lung carcinoma (LLC) and NSCLC cells in vitro proliferation, invasion, and in vitro soft agar colony formation were assessed after treating them with MNTX or shRNA MOR. Also, in vivo primary tumor growth and lung metastasis were assessed in C57BL/6 and MOR knockout mice. Treatment with MNTX or silencing MOR expression inhibited LLC invasion and anchor-independent growth. Injection of MOR-silenced LLC leads to a reduction in mouse lung metastasis. Furthermore, MOR knockout mice do not develop significant tumors when injected with LLC compared to wild-type controls. Finally, continuous infusion of the peripheral opioid antagonist MNTX attenuates primary LLC tumor growth and reduces lung metastasis [18].

Tumor cell latency is a major problem in chemotherapy as it limits the therapeutic efficacy of antitumor drugs that only target actively dividing cells. One possible way to overcome chemotherapy resistance is to activate dormant cells. The MNTX can have a beneficial effect since it enhances the effect of docetaxel by blocking a cell growth suppressive pathway [44]. MNTX blocks OGF signaling to free cancerous cells from their arrest, thereby increasing docetaxel therapeutic efficacy. Combining docetaxel and MNTX significantly prolongs survival, relieves abdominal pain, decreases abdominal pain, and diminishes docetaxel resistant spheroids on the peritoneal membrane inhibiting micrometastasis formation and increasing survival time in a murine model of gastric cancer with peritoneal dissemination [48].

Endogenous opioids decrease human ovarian cancer cell proliferation. One study found that NTX alone or combined with standard therapies, i.e., taxol/paclitaxel or cisplatin, altered human ovarian cancer cell proliferation in tissue culture and tumor progression in a murine model. Administration of low-dose NTX for six hours every other day, but not continuously, reduced DNA synthesis and cell replication of vehicle-treated controls in tissue culture. Furthermore, short exposure to NTX in combination with taxol or cisplatin had enhanced anticancer effect. Mice with established ovarian tumors treated with low-dose NTX have minor tumor progression by reducing DNA synthesis and angiogenesis without altering cell survival. The combination of low-dose NTX with cisplatin, but not with taxol, resulted in an additive inhibitory effect on tumorigenesis with further depression of DNA synthesis and angiogenesis [38]. Another study investigated the impact of upregulation of the OGF-OGFr axis by OGF or low-dose NTX treatment on human ovarian tumorigenesis in vivo. Female nude mice were intraperitoneally transplanted with SKOV-3 human ovarian cancer cells and treated daily with OGF, low-dose NTX, or an equivalent saline placebo. Tumor burden, DNA synthesis, apoptosis, and angiogenesis in tumor tissue were evaluated after 40 days of treatment. Authors found that OGF and low-dose NTX markedly reduced the ovarian tumor burden, i.e., the number and weight of tumor nodules. The mechanism of action was directed at inhibiting tumor cell proliferation and angiogenesis; no changes in cell survival were observed [39].

One study investigated the modulation of the OGF–OGFr axis by (1) exogenous OGF, (2) upregulation of OGFr using imiquimod, or (3) intermittent opioid receptor blockade with a low dose of NTX on the progression of established squamous cell carcinoma of the head and neck (SCCHN). Nude mice with visible human SCCHN SCC-1 tumors received (1) OGF or low-dose NTX or (2) imiquimod. Tumor growth and DNA synthesis were monitored. OGF and low-dose NTX increased the latency from visible to measurable tumors up to 1.6-fold. OGF, low-dose NTX, and imiquimod treatment reduced tumor volume and weight, and decreased DNA synthesis in tumors [49].

NTX had an inhibitory effect on S2OY neuroblastoma (NB) growth in A/Jax mice. Daily injections of 0.1 mg/kg NTX resulted in prolonged time-to-tumor appearance and an increase in median survival time. The pattern and incidence of metastases of NTX and control mice were similar [50].

Regarding other types of MOR antagonists, one study investigated whether nalmefene could inhibit CT26 CRC cells growth by influencing cellular glycolysis. The authors conclude that the antitumor effect of nalmefene can be achieved by, calmodulin, and serine/threonine kinase (AKT) -glycogen synthase kinase -3β (GSK-3β) pathway inhibition [33].

We also found publications where MOR antagonists antitumor role is questioned, especially if using NTX at high and continuous doses. A study was conducted to investigate the effects of successive treatment with clinically relevant doses of NTX on human T24 and murine MB49 bladder cancer cells proliferation, migration, and invasion capability. The results showed that successive NTX treatment significantly promoted proliferation, decreased apoptosis of bladder cancer cells, and increased cell migration and invasiveness. Continuous NTX treatment also significantly reduced the expression of epithelial markers, i.e., E-cadherin and cytokeratin 19, increasing mesenchymal markers' expression, i.e., N-cadherin and vimentin EMT-inducing Snail and Slug transcription factors. The PI3K/AKT signalling pathway was activated by successive NTX treatments. Thus, these results suggest that treatment with NTX may favor the progression of bladder tumors [51].

### Clinical Evidence

Data regarding MOR antagonists usage for cancer treatment in the clinical setting are scarce. Apart from one unplanned post hoc pooled analysis on data from two phase III and IV randomized clinical trials (RCTs) on MTNX [52•] and one randomized crossover preliminary trial, [53] the literature mainly consists of case reports or case series where low-dose NTX, together with other types of drugs, shows an improvement in oncological prognosis.

The above-mentioned pooled data analysis was carried out combining data from two RCTs analyzing the effect of MNTX compared to placebo on OIC despite laxatives in patients with advanced end-stage cancer. The authors assessed the effect of MNTX on overall survival (OS) and found that MNTX treatment, especially if yielding a clinical response on OIC, was associated with longer OS [52•].

NTX has been used alone[54] or with adjuvants in several case reports where prolonged survival in patients with advanced-stage cancer has been reported. Vitamin C and D, [55] alpha-lipoic acid (ALA) both with, [56, 57] or previous to NTX administration [58] and vitamin C plus ALA [59] have been used as adjuvants in various types of cancers. The effect seems to be linked to oxidative stress reduction, proapoptotic effect, and proliferation inhibition.

Furthermore, two studies focused on neuroendocrine system immunomodulatory effect in metastatic cancer patients. One preliminary randomized crossover study was conducted to obtain preliminary results on NTX inhibition of brain opioids in humans. Ten patients with metastatic renal cancer were treated with an IL-2 and NTX combination, and the majority showed decreased disease progression [53]. The second cases series report reported on a partial benefit in disease progression showed by a combined ALA and NTX treatment [60].

## Conclusions

We found consistent preclinical evidence of a potential influence of MOR antagonists (naltrexone and methylnaltrexone) on cancer growth and spread mediated by an effect on both host and cancer cells. Clinical results are scarce and limited to poor-quality evidence. Further high-quality studies such as randomized clinical trials are warranted to study the potential role of MOR antagonists as a therapeutic alternative in different types of cancer, especially in patients where the standard treatment causes unacceptable side effects. Low doses of naltrexone could be a therapeutic option in the perioperative period, while methylnaltrexone could be used later as an adjuvant to numerous chemotherapy drugs.
